# Static and Dynamic Cognitive Reserve Proxy Measures: Interactions with Alzheimer’s Disease Neuropathology and Cognition

**DOI:** 10.4172/2161-0460.1000390

**Published:** 2017-10-25

**Authors:** Michael Malek-Ahmadi, Sophie Lu, YanYan Chan, Sylvia E Perez, Kewei Chen, Elliott J Mufson

**Affiliations:** 1 Banner Alzheimer’s Institute 901 E. Willetta St. Phoenix, AZ, USA; 2 Williams College, Williamstown, MA, USA; 3 Arizona State University, Tempe, AZ, USA; 4 Department of Neurobiology, Barrow Neurological Institute, 350 W. Thomas Rd. Phoenix, AZ, USA

**Keywords:** No cognitive impairment, Mild cognitive impairment, Episodic memory, Executive function, Plaque, Tangle, Education, Intellectual function

## Abstract

**Objective:**

Years of education are the most common proxy for measuring cognitive reserve (CR) when assessing the relationship between Alzheimer’s disease (AD) neuropathology and cognition. However, years of education may be limited as a CR proxy given that it represents a specific timeframe in early life and is static. Studies suggest that measures of intellectual function provide a dynamic estimate of CR that is superior to years of education since it captures the effect of continued learning over time. The present study determined whether dynamic measures of CR were better predictors of episodic memory and executive function in the presence of AD pathology than a static measure of CR.

**Methods:**

Subjects examined died with a pre-mortem clinical diagnosis of no cognitive impaired, mild cognitive impairment and mild to moderate AD. CERAD and Braak stage were used to stratify the sample by AD pathology severity. Linear regression analyses using CR by CERAD and CR by Braak stage interaction terms were used to determine whether Extended Range Vocabulary Test (ERVT) scores or years of education were significantly associated with episodic memory composite (EMC) and executive function composite (EFC) performance. All models were adjusted for clinical diagnosis, age at death, gender, APOE e4 carrier status and Braak stage.

**Results:**

For episodic memory, years of education by CERAD interaction were not statistically significant (β=-0.01, SE=0.01, p=0.53). By contrast, ERVT interaction with CERAD diagnosis was statistically significant (β=-0.03, SE=0.01, p=0.004). Among the models using Braak stages, none of the CR by pathology interactions were associated with EMC or EFC.

**Conclusion:**

Results suggest that a dynamic rather than a static measure is a better indicator of CR and that the relationship between CR and cognition is dependent upon the severity of select AD criteria.

## Introduction

Cognitive reserve (CR) refers to an individual’s ability to maintain cognitive function in the presence of damaging brain pathology [1]. In the context of Alzheimer’s disease (AD), CR has received a great deal of attention given the increasing interest in establishing associations between cognition and AD pathology. During the past several years there has been a shift towards identifying and studying individuals in the pre-clinical stages of AD. Therefore, there is a need to accurately characterize and quantify CR as a risk factor for the progression from no cognitive impairment (NCI) to mild cognitive impairment (MCI).

Although higher scores on CR proxy measures are generally associated with better cognitive performance [2], the neural mechanisms underlying this association are not fully understood. Evidence from neuroimaging studies [3-5] supports the concepts of brain reserve and neural compensation as possible mechanisms underlying CR [6]. Brain reserve suggests that greater efficiency and capacity of networks involved in cognition allow individuals to maintain greater levels of cognitive function despite the presence of deleterious pathologies [2]. Neural compensation posits that additional brain networks are recruited to maintain cognitive function when previously established networks are degraded by neuropathology [2]. Resting-state fMRI investigations provide evidence that greater CR were associated with superior functional connectivity and higher efficiency in several cortical regions [7]. This study revealed that MCI individuals with higher CR showed enhanced network connectivity in fronto-parietal and decreased connectivity in fronto-temporo-cerebellar nodes, but not in NCI and AD subjects [7].The latter finding suggests that the protective effect of CR occurs during the prodromal phase(s) of AD [7], which is mitigated by increased neuropathological burden during disease progression.

Moreover, CR was found to moderate the association between hippocampal volume and episodic memory, but a significant association between hippocampal volume and episodic memory was not reported in NCI individuals [7]. Education, a component of CR, interacted with neuritic plaques (NPs) and dementia, but not with diffuse plaques (DPs) or neurofibrillary tangles (NFTs) [8].

Since the mechanisms underlying the concept of CR are not directly measurable, proxy determinants relating brain resilience to neuropathology and cognition require continued investigation [2]. Although the most common CR proxy is the number of years of formal education [8,9], measures of intelligence [10], occupational attainment [11], level of literacy [12] and engagement in cognitively based activities [13] have been employed. Variables that influence CR can be characterized as either static or dynamic measures. Occupational attainment and years of education usually remain static after a certain time whereas literacy, engagement in cognitive activities, and general intellectual function are dynamic constructs likely to be altered over time. As a result, the static versus dynamic characterizations of CR may have different effects on the interaction between cognitive decline and AD neuropathology.

It is possible that dynamic measures reflect enrichment of CR that occurs beyond occupational and educational attainment, providing a better estimate of CR. In the present study, we investigated whether 1) CR is better characterized by static or dynamic measures and 2) whether interactions between static and dynamic measures of CR and AD neuropathology affect cognition.

## Methods

### Study sample

Data examined was derived from 249 older deceased persons who were participants in the Rush Religious Orders Study (RROS) [14,15] that died with a pre-mortem clinical diagnosis of no cognitive impairment (NCI; n=123), MCI (n=79) and AD (n=47), that had no coexisting clinical or neurological conditions judged to contribute to cognitive impairment at their last clinical evaluation [16,17], agreed to an annual clinical evaluation and signed an informed consent and an Anatomic Gift Act donating their brains at time of death. Data from these subjects have been used in numerous clinical pathological studies supported by our ongoing NIA program project grant entitled the “Neurobiology of Mild Cognitive Impairment in the Elderly” (PO1AG14449). At the time of these studies, individuals were chosen from all available RROS participants that came to autopsy during a rolling admission [16]. Subjects that were receiving anticholinesterases or medication for depression were excluded.The Human Investigation Committee of Rush University Medical Center approved this study.

### Clinical evaluation

Participants underwent a uniform and structured clinical evaluation obtained by a team led by a neurologist, and cognitive function was determined by a trained neuropsychological test technician [15,16]. Medications used by the subjects within the previous fourteen days of the examination were reviewed and classified. After review of all clinical data and examination of the participant, a clinical diagnosis was made by a board certified neurologist or geriatrician with expertise in the evaluation of elderly persons with dementia. A neurologist reviewed the medical history, medication use, neurologic examination, results of cognitive performance testing and the neuropsychologist’s opinion of cognitive impairment and dementia. Each participant was evaluated in their home, emphasizing findings deemed clinically relevant. Clinical diagnostic classification was performed as described previously [15,16].

### Tissue preparation and neuropathological diagnosis

Brain accruement and processing was described previously [17,18]. Briefly, each brain was cut into 1 cm thick coronal slabs using a brain slice apparatus and hemisected. One hemisphere was immersion fixed in 4% paraformaldehyde (24-72 h) and cryoprotected (10% glycerol and 2% dimethyl sulfoxide in phosphate buffer solution) until processing for immunohistochemistry and the other flash frozen.

Diagnostic blocks (mid-frontal, superior temporal, entorhinal cortex, hippocampus, inferior parietal cortex, basal ganglia, thalamus and substantia nigra) from the opposite hemisphere were paraffin embedded and sectioned at 6 μm. Examination for cerebral infarctions was conducted as described previously [19]. Bielschowsky silver stain was used to visualize NPs, DPs and NFTs. Sections were also immunostained with the Aβ M0872 antibody (1:100; Dako, CA) rose against Aβ_1-40_ and Aβ_1-42_. NFTs were also visualized using a paired helical filament tau antibody (AT8; 1:800, Covance). Neuropathological diagnoses were determined according to CERAD [20] and Braak staging [21] as recommended by the NIA-Reagan criteria [22]. Exclusion criteria included mixed dementias, Parkinson’s disease, frontotemporal dementia, argyrophilic grain disease, vascular dementia, hippocampal sclerosis, stroke and Lewy body disease. Lewy bodies (LB) in the substantia nigra, entorhinal, cingulate, midfrontal, middle temporal and inferior parietal cortex were detected using α-synuclein (αSyn) immunohistochemistry as previously described [23]. Depending upon the distribution of the αSyn-positive tissue was scored semi-quantitatively according to the severity and anatomical distribution, separating brainstem predominant (PD), limbic/ transitional and difuse neocortical subtypes, [24,25]. A board-certified neuropathologist or trained technician, blinded to clinical diagnosis, counted number of NPs, DPs revealed by Bielschowsky silver stain and immunohistochemistry using the phosphorylated paired helical filament tau AT8 marker for NFTs, respectively, in one square mm area (100x magnification) per cortical region as reported previously [16,26].

### Cognitive composite scores

An Episodic Memory Composite (EMC) score was derived from the results of the following tests: WMS-R Logical Memory Story, Immediate and Delayed Recall, CERAD Word List Immediate Recall, CERAD Word List Delayed Recall, CERAD Word List Recognition. The Executive Function Composite (EFC) used the Symbol Digit Modalities Test, Category Fluency Test, and Ravens Progressive Matrices. Composite scores were first derived by converting raw test scores into z-scores using the sample mean and sample standard deviation from the NCI group for each test.The resulting z-scores from each test were averaged to create composite scores. Cognitive composite score data was derived from the last clinic visit prior to autopsy.

### Cognitive reserve proxy measures

Proxy measures of CR included years of completed formal education and the Extended Range Vocabulary Test (ERVT) [27]. ERVT is a 15-item test that requires participants to identify the correct synonym for a word from a group of five other words. Years of formal education completed were obtained via self-report from each participant. For this analysis, the baseline ERVT was used in order to provide the best estimate of pre-morbid CR as all subjects were cognitively normal at study entry.

### Statistical Analysis

For demographic and cognitive variables, one-way analysis of variance (ANOVA) test was used to analyze group differences for continuous variables. Significant group wise comparisons employed the Tukey HSD test. Chi-square test was used to analyze differences in categorical variables.

Linear regression models were used to assess whether years of education and ERVT score were significant predictors of EMC and EFC. For each model, clinical diagnosis, age at death, gender, and APOE ε4 carrier status were included to account for their effects. CERAD diagnosis and Braak stage were assessed in separate models with each of the CR measures.The models also included interaction terms for CERAD with CR and Braak stage with CR. CERAD diagnosis was coded numerically using the following scheme: 1=No AD, 2=Possible AD, 3=Probable AD, 4=Definite AD. Braak stage was collapsed into three categories and coded numerically: 1=Braak Stage 0-II, 2=Braak Stage III, 3=Braak Stage IV-V. Adjusted R^2^ and Akaike’s Information Criteria (AIC) values were derived to assess model fit. Linear relationships between the CR and cognitive measures were confirmed by a comparison of linear and quadratic fits where the AIC values did not differ substantially between these models.

Statistical significance was set at p ≤ 0.05. Groupwise comparisons of demographic, cognitive, and neuropathological variables were carried out using SYSTAT 13.0. Linear regression analyses were carried out with R 3.4.0 using the ‘lm’ function.

## Results

The cohort examined was comprised of 109 males and 140 females with an average age at death of 85.21 ± 5.91 years and a mean of 18.26 ± 3.39 years of education.The average duration between last clinical assessment and autopsy was 0.73 ± 0.67 years and average post-mortem interval (PMI) was 7.63 ± 7.01 h. Descriptive statistics by clinical group are shown in Table 1. No significant differences in gender frequency were noted between the groups while the APOE ε4 allele was most prevalent in the AD cases (p=0.04). Education levels were similar between groups (p=0.86) while AD and MCI had a significantly higher age at death compared to NCI cases (p<0.001). PMI and brain weight at autopsy showed no significant between-group differences (p=0.66, p=0.09, respectively).The duration between the last clinical assessment and autopsy did not differ between clinical groups (p=0.97).

The NCI group had a significantly greater percentage (37%) of low Braak stages (0, I, II) compared to the MCI (22%) and AD (9%) groups.The prevalence of Braak stage III was similar across groups (NCI-28%, MCI-24%, AD-23%) while more advanced Braak stages (IV and V) were predominant in the AD group (68%) followed by MCI (54%) and NCI (35%); (p<0.001).The NCI group contained a greater proportion of individuals with a CERAD classification of ‘No AD’ compared to MCI and AD. For NIA Reagan diagnosis, the classifications of ‘Not AD’ and ‘Low Likelihood’ occurred more frequently in NCI compared to the MCI and AD groups (Table 1). Descriptive statistics for the MMSE, ERVT, EMC and EFC are shown in Table 2. All measures examined displayed significant differences (NCI>MCI>AD, p<0.001). Skewness values for the EMC and EFC were -0.45 and 0.33, respectively, indicating that both variables met the assumption of normality.

**Figure 1:**
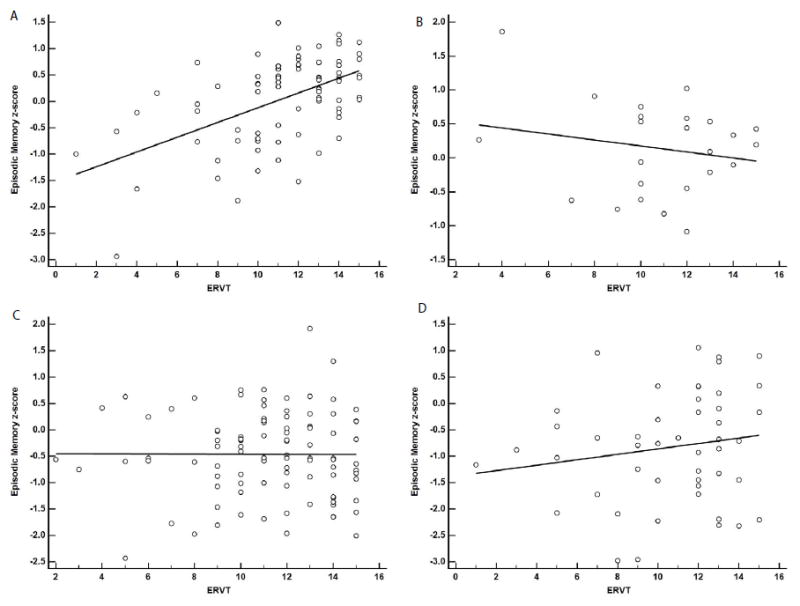
Linear regression analysis showing association between ERVT score and episodic memory and CERAD neuropathological diagnostic groups. A-No AD (β=0.14, SE=0.02, p<0.001), B-Possible AD (β=0.04, SE=0.05, p=0.36), C-Probable AD (β=0.00, SE=0.03, p=0.97), D-Definite AD (β=0.05, SE=0.05, p=0.26)

Results of the linear regression models using years of education and ERVT scores to predict EMC and EFC scores are shown in Tables 3A and 3B. For the EMC, years of education by CERAD interaction were not statistically significant (β=-0.01, SE=0.01, p=0.53). For this model the adjusted R^2^ was 0.58 and the AIC value was 450.06. By contrast, the ERVT and its interaction with CERAD diagnosis was statistically significant (β=-0.03, SE=0.01, p=0.004) (Figure 1). The adjusted R^2^ was 0.59, while the AIC value was 443.97 indicating a slightly better fit for the ERVT model. A breakdown of the ERVT by CERAD interaction found that the effect was likely driven by the positive association in the No AD pathology group (β=0.14, SE=0.02, p<0.001; Figure 1A) as slopes for the other CERAD groups were not significantly different [Possible AD (β=-0.04, SE=0.05, p=0.36; Figure 1B); Probable AD (β=0.00, SE=0.03, p=0.97; Figure 1C); Definite AD (β=0.05, SE=0.05, p=0.26; Figure 1D)].

For EMC, the interaction of years of education and Braak stage (β=0.01, SE=0.01, p=0.66) was also not statistically significant.The adjusted R^2^ was 0.59 and the AIC value was 447.69.The ERVT and Braak stage interaction (β=0.02, SE=0.01, p=0.20) also showed non-significant results with an adjusted R^2^ value 0.59 and an AIC value of 448.24 (Table 3B).

**Figure 2:**
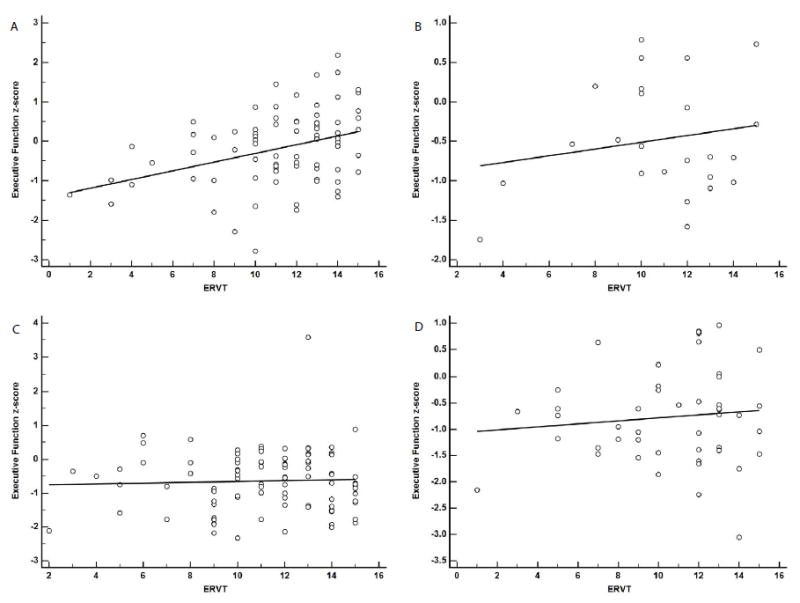
Linear regression analysis showing association between ERVT score and executive function and CERAD diagnostic groups. A-No AD (β=0.11, SE=0.03, p<0.001), B-Possible AD (β=0.04, SE=0.05, p=0.39), C-Probable AD (β=0.01, SE=0.03, p=0.70), D-Definite AD (β=0.03, SE=0.04, p=0.

For the EFC, the interaction for years of education and CERAD (β=-0.01, SE=0.01, p=0.39) was not statistically significant.The adjusted R^2^ was 0.47 and the AIC value was 512.40 for this model. In contrast, the ERVT and CERAD interaction (β=-0.03, SE=0.01, p=0.01) was statistically significant (Figure 2). A breakdown of the ERVT by CERAD interaction found that the effect was likely driven by the positive association in the No AD pathology group (β=0.11, SE=0.03, p<0.001; Figure 2A) as the slopes for the other CERAD groups were not statistically significant [Possible AD (β=0.04, SE=0.05, p=0.39; Figure 2B); Probable AD (β=0.01, SE=0.03, p=0.70; Figure 2C); Definite AD (β=0.03, SE=0.04, p=0.46; Figure 2D)].The adjusted R^2^ was 0.48, while the AIC value was 506.34 indicating a slightly better fit for the ERVT model.

For the EFC, the interaction of years of education and Braak stage (β=-0.01, SE=0.02, p=0.33) was not statistically significant.The adjusted R^2^ was 0.47 and the AIC value was 512.86.The ERVT and Braak stage interaction was not significant (β=-0.03, SE=0.02, p=0.09). For the ERVT and the Braak model, the adjusted R^2^ was 0.47 and the AIC value was 510.68 indicating a slightly better fit relative to the years of education model (Table 3B).

## Discussion

The results of this study found that both episodic memory and executive function performance are impacted by the interaction between CR and AD neuropathology. Specifically, there was a positive association between ERVT and cognition among individuals with lower plaque pathology (CERAD-No AD). Among the CERAD groups that had greater AD neuropathology, the slopes for the CR and cognition association were less steep and in some cases nearly flat suggesting that the protective effects of CR are most pronounced in the early stages of AD pathogenesis. These results also suggest that when the accumulation of AD pathology reaches a certain threshold the benefits of CR are negated. None of the models using years of education as a CR proxy showed significant associations with cognition while ERVT and its interaction with AD pathology showed significant associations with episodic memory and executive function. This suggests that dynamic CR measures are superior to static CR measures in their ability to predict cognitive performance in the presence of AD pathology. Furthermore, the differences in AIC values, which favored the ERVT models over the years of education models, provide evidence supporting the use of CR proxy measures, which may indicate that development of dynamic CR occurs beyond the completion of formal education.

Schwartz et al. [28] indicated that using years of education as a CR proxy is problematic because it ignores cognitively enriching activities that occur both prior to and after the completion of formal education. In addition, years of education may also be limited because it conceptualizes CR as a function of academic intelligence and ignores other types of intelligence [28]. Others have suggested that using education level as a CR proxy can vary substantially based on the methods used to determine high and low levels of education [29] raising uncertainty about the strength of the association between years of education and cognition. It has been suggested that engagement in cognitively-based leisure activities in adulthood are a better measure of CR than years of education and that the build-up of CR is a fluid process that occurs throughout an individual’s lifetime [30].

The significant interactions between the ERVT and CERAD diagnosis on cognition found here suggest that intellectually-based CR measures provide a more reliable measure of CR than educational attainment, since the effect of continued learning over a lifetime might be better taken into account. This is supported by findings showing that the interaction of performance on the American National Reading Test (AMNART) and amyloid deposition was significantly associated with cognitive performance in NCI individuals [31]. A recent study found that NCI, MCI, and AD subjects with higher AMNART performance had higher MMSE scores even in the presence of significant NFT pathology [32]. Although our results did not demonstrate a significant interaction of CR and NFT pathology (Braak stage), we did show that higher ERVT scores were associated with better executive function performance after accounting for the interaction of Braak stage. This result is similar to other findings showing that variability in cognitive performance among NCI individuals is associated with AMNART performance and that this association is independent of AD pathology [33]. It was also reported that the AMNART’s impact on cognition was additive, rather than dependent, in the context of AD pathology [33].

Although our results show that the performance of dynamic CR proxies are superior to static ones, their associations with cognition are likely influenced by a number of other neuroprotective factors that underlie CR. For example, engagement in cultural, spiritual, physical, intellectual, and communal activities may contribute to CR development [28] suggesting that it is determined by multiple factors rather than a single factor. Others have created a questionnaire that integrates the quantity and duration of various cognitive and lifestyle activities to form a CR index [34], however a search of the literature did not yield any studies that tested this instrument’s interaction with AD pathology and cognition. Perhaps studies using this index [34] would yield stronger associations between CR, AD pathology and cognition.

With AD research shifting its focus toward prevention trials in asymptomatic individuals [35,36], including a measure of CR may be important for defining the trajectory of cognitive changes. Since increased CR mitigates the negative effect of AD neuropathology on cognition, differing levels of CR among individuals in the placebo and treatment groups in clinical trials may adversely impact the ability to detect a significant treatment effect [37]. Since effect sizes for cognitive tests are often small [38] and many clinical trials have shown a lack of decline in their placebo groups [39], accounting for CR in the design of AD prevention trials may help detect significant treatment effects. Recent evidence indicates that utilizing Braak staging increases the ability to detect longitudinal differences in ante-mortem cognitive performance [40], which may be complimented by *in vivo* tau imaging [41]. Perhaps utilizing tau imaging in conjunction with CR measures would reduce the sample size needed for AD prevention trials. However, previous findings have shown that NCI individuals can display AD pathology similar to those with clinical MCI and AD [42,43], suggesting that these individual have high levels of CR. How this effects subject selection for clinical trials and its use with tau imaging remains to be determined.

## Conclusion

A limitation to this study was its cross-sectional design, which limits its prognostic value. In addition, our subjects were from a community-based group of highly educated retired clergy who had excellent health care and nutrition. Individuals who volunteer may introduce bias by decreasing pathology but this is partially mitigated by high follow-up and autopsy rates [19]. Another limitation is the small number of APOE ε4 carriers, particularly homozygous individuals. Future studies with a greater balance of APOE ε4 carriers and non-carriers will extend these results. Strengths include uniform pre-mortem clinical and postmortem pathological evaluation and that final pathologic classification was performed without knowledge of clinical evaluation. Despite these caveats, we found that dynamic, compared to static proxies of CR are superior in terms of predicting cognitive performance in the presence of AD pathology.

## Figures and Tables

**Table 1:** Demographic and neuropathological characteristics for NCI, MCI and AD.

	NCI	MCI	AD	p-value	Group Differences

N	123	79	47	------	

Gender (M/F)	63/60	29/50	17/30	0.06	

APOE ε4 (Carrier/Non-Carrier)	21/101*	20/57*	16/30*	0.04	

Age at Death	83.90 ± 6.12	85.97 ± 5.57	87.38 ± 5.10	<0.001	NCI<MCI, NCI<AD, MCI=AD (p=0.38)

Education (years)	18.29 ± 3.59	18.35 ± 3.38	18.02 ± 2.89	0.86	

Time Between Last Clinic Visit and Autopsy (years)	0.73 ± 0.79	0.74 ± 0.58	0.71 ± 0.43	0.97	

Post-Mortem Interval (h)	7.58 ± 7.40	8.12 ± 7.24	6.94 ± 5.46	0.66	

Brain Weight (g)	1,237.97 ± 149.12	1,215.62 ± 159.60	1,183.16 ± 96.97	0.09	

**CERAD Diagnosis**				<0.001	
No AD	50	18	5		
Possible AD	16	17	1		
Probable AD	44	18	23		
Definite AD	13	26	18		

**NIA Reagan Diagnosis**				<0.001	
Not AD	3	4	0		
Low Likelihood	69	29	7		
Intermediate Likelihood	49	42	29		
High Likelihood	2	4	11		

**Braak Stage**				<0.001	
0-II	45	17	4		
III	35	19	11		
IV-V	43	43	32		

* APOE genotype not available for one individual in NCI and AD, two in MCI; mean ± standard deviation

**Table 2:** MMSE, cognitive reserve, and cognitive domain statistics for NCI, MCI and AD.

	NCI	MCI	AD	p-value	Group Differences
MMSE	28.29 ± 1.37	26.73 ± 2.43	23.34 ± 2.30	<0.001	NCI>MCI>AD
ERVT	11.58 ± 2.84	10.19 ± 3.27	8.47 ± 3.56	<0.001	NCI>MCI>AD
EMC z-score	0.31 ± 0.51	-0.72 ± 0.41	-1.50 ± 0.71	<0.001	NCI>MCI>AD
EFC z-score	0.00 ± 0.77	-0.56 ± 0.78	-1.50 ± 0.58	<0.001	NCI>MCI>AD

**Table 3:** 

A: Linear regression model results for cognitive reserve and CERAD interactions as predictors of cognition.				
	Episodic Memory		Executive Function	

	Slope (SE)	p-value	Slope (SE)	p-value

Education × CERAD	-0.006 (0.01)	0.53	-0.01 (0.01)	0.39

	Adjusted R^2^=0.58		Adjusted R^2^=0.47	
	AIC=450.06		AIC=512.40	

	**Slope (SE)**	**p-value**	**Slope (SE)**	**p-value**

ERVT × CERAD	-0.03 (0.01)	0.004	-0.03 (0.01)	0.01

	Adjusted R^2^=0.59		Adjusted R^2^=0.48	
	AIC=443.97		AIC=506.34	

**B:** Linear regression model results for cognitive reserve measures and Braak stage as predictors of cognition.				
	**Slope (SE)**	**p-value**	**Slope (SE)**	**p-value**

Education × Braak Stage	0.006 (0.01)	0.66	-0.02 (0.02)	0.33

	Adjusted R^2^=0.59		Adjusted R^2^=0.47	
	AIC=447.69		AIC=512.86	

	**Slope (SE)**	**p-value**	**Slope (SE)**	**p-value**

ERVT × Braak Stage	0.02 (0.01)	0.20	-0.03 (0.02)	0.09

	Adjusted R^2^=0.59		Adjusted R^2^=0.47	
	AIC=448.24		AIC=510.68	

SE: Standard Error; AIC: Akaike’s Information Criteria

All models adjusted for clinical diagnosis, age at death, gender and APOE ε4 carrier status

SE: Standard Error; AIC: Akaike’s Information Criteria

CAll analyses adjusted for clinical diagnosis, age at death, gender and APOE ε4 carrier status
